# Preclinical Characteristics of the Irreversible Pan-HER Kinase Inhibitor Neratinib Compared with Lapatinib: Implications for the Treatment of HER2-Positive and *HER2*-Mutated Breast Cancer

**DOI:** 10.3390/cancers11060737

**Published:** 2019-05-28

**Authors:** Denis M. Collins, Neil T. Conlon, Srinivasaraghavan Kannan, Chandra S. Verma, Lisa D. Eli, Alshad S. Lalani, John Crown

**Affiliations:** 1 National Institute for Cellular Biotechnology, Dublin City University, Glasnevin, 9 Dublin, Ireland; neil.conlon@dcu.ie (N.T.C.); john.crown@ccrt.ie (J.C.); 2 Bioinformatics Institute, A*STAR (Agency for Science, Technology and Research), 30 Biopolis Street, #07-01 Matrix, Singapore 138671, Singapore; raghavk@bii.a-star.edu.sg (S.K.); chandra@bii.a-star.edu.sg (C.S.V.); 3 Department of Biological Sciences, National University of Singapore, 14 Science Drive 4, Singapore 117543, Singapore; 4 School of Biological Sciences, Nanyang Technological University, 50 Nanyang Drive, Singapore 637551, Singapore; 5 Puma Biotechnology, Inc., 10880 Wilshire Blvd., Suite 2150, Los Angeles, CA 90024, USA; leli@pumabiotechnology.com (L.D.E.); ALalani@pumabiotechnology.com (A.S.L.); 6 Department of Medical Oncology, St Vincent’s University Hospital, 4 Dublin, Ireland

**Keywords:** tyrosine kinase inhibitors, lapatinib, neratinib, HER2, breast cancer

## Abstract

An estimated 15–20% of breast cancers overexpress human epidermal growth factor receptor 2 (HER2/ERBB2/neu). Two small-molecule tyrosine kinase inhibitors (TKIs), lapatinib and neratinib, have been approved for the treatment of HER2-positive (HER2+) breast cancer. Lapatinib, a reversible epidermal growth factor receptor (EGFR/ERBB1/HER1) and HER2 TKI, is used for the treatment of advanced HER2+ breast cancer in combination with capecitabine, in combination with trastuzumab in patients with hormone receptor-negative metastatic breast cancer, and in combination with an aromatase inhibitor for the first-line treatment of HER2+ breast cancer. Neratinib, a next-generation, irreversible pan-HER TKI, is used in the US for extended adjuvant treatment of adult patients with early-stage HER2+ breast cancer following 1 year of trastuzumab. In Europe, neratinib is used in the extended adjuvant treatment of adult patients with early-stage hormone receptor-positive HER2+ breast cancer who are less than 1 year from the completion of prior adjuvant trastuzumab-based therapy. Preclinical studies have shown that these agents have distinct properties that may impact their clinical activity. This review describes the preclinical characterization of lapatinib and neratinib, with a focus on the differences between these two agents that may have implications for patient management.

## 1. Introduction

Five human epidermal growth factor receptor 2 (HER2/ERBB2/neu)-targeted therapies are currently approved by the Food and Drug Administration (FDA) for the treatment of HER2-positive (HER2+) breast cancers: trastuzumab, pertuzumab, trastuzumab emtansine (T-DM1), lapatinib, and neratinib [1]. These can be divided into three categories: anti-HER2 monoclonal antibodies (trastuzumab and pertuzumab), antibody–drug conjugate (T-DM1), and small-molecule pan-HER tyrosine kinase inhibitors (TKIs; lapatinib and neratinib). A number of other HER-directed TKIs are currently in development, including tucatinib [2], poziotinib [3], and pyrotinib [4]. This review focuses on preclinical findings regarding lapatinib and neratinib, the two approved small-molecule pan-HER TKIs, in order to provide an in-depth and comprehensive review of preclinical data. Clinical trials involving lapatinib and neratinib have been reviewed elsewhere [5,6] and are not covered in this review.

Despite their similarities, multiple distinguishing characteristics between lapatinib and neratinib have been reported (Table 1). The two most striking are (1) the nature of target binding: lapatinib binds reversibly whereas neratinib binds irreversibly, and (2) differential affinities for epidermal growth factor receptor (EGFR/HER1/ERBB1), HER2, and HER4 (ERBB4). Additional differentiators include kinome profiles, in vitro cytotoxicities, effects on HER-family dimerization, effects on HER2 endocytosis, inhibition of mutant EGFR, HER2, or HER4, and resistance mechanisms.

## 2. The HER/c-ERBB Family

The HER family is a group of receptor tyrosine kinases (RTKs) associated with tumorigenesis [15]. The family has four members: EGFR, HER2, HER3 (ERBB3), and HER4 [15,16]. These receptors are ubiquitously expressed and are critical for normal development. HER-family RTKs have a common structural organization consisting of a ligand-binding extracellular domain (ECD), a single transmembrane segment, and an intracellular protein kinase domain with a carboxyterminal tail [17]. The ECD comprises four subdomains. Subdomain I, when bound to ligand, binds to subdomain III [18], triggering a conformational change that exposes subdomain II, the dimerization domain, allowing for receptor homo- or heterodimerization and kinase activation. Domains II and IV are involved in disulfide bond formation. Trans- or auto-phosphorylation of the C-terminal tyrosine-bearing motifs in the tail of HER family members [15,19] enables recruitment of signaling molecules with Src homology 2 and phosphotyrosine-binding domains, and initiation of intracellular signaling cascades including the mitogen-activated protein kinase (MAPK) and phosphoinositide 3-kinase (PI3K) pathways [20]. These pathways subsequently activate transcription factors affecting cell survival, proliferation, motility, and differentiation [15,17,19].

**Figure 1:**
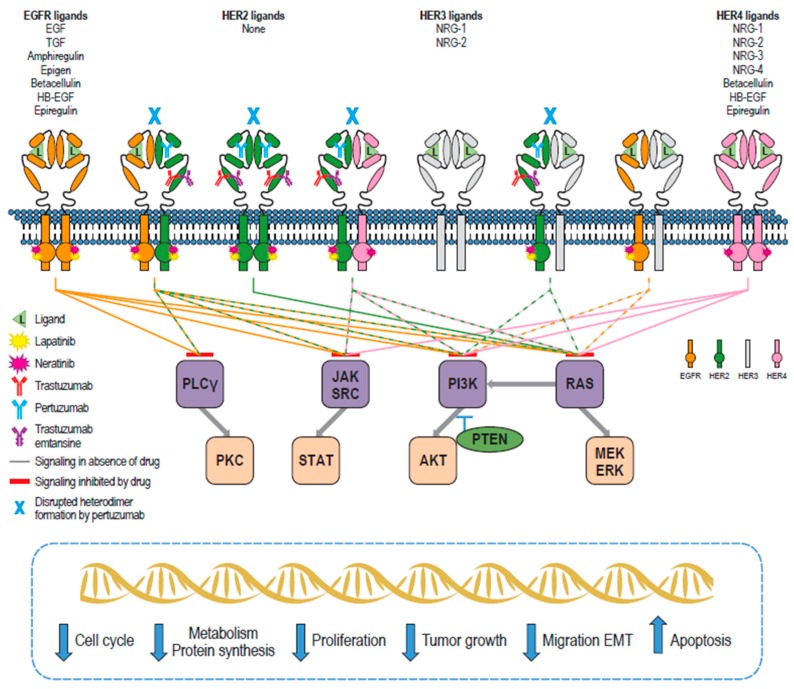
Schematic representation of members of the human epidermal growth factor receptor (HER) family, their natural ligands, interaction with the HER2-targeted therapies trastuzumab, pertuzumab, trastuzumab emtansine (T-DM1), lapatinib, and neratinib, and the downstream consequences of these interactions. Circles on EGFR, HER2, and HER4 represent the active kinase domain; the kinase domain on HER3 is inactive. Ligand binding initiates homo- or heterodimerization of HER family members, leading to intracellular signaling cascades that are dependent on the dimers formed (e.g., PI3K/AKT and MAPK [Ras/MEK/extracellular signal-regulated kinase (ERK)]). HER-family signaling affects multiple cell processes including cell cycle, metabolism/protein synthesis, proliferation, migration, epithelial–mesenchymal transition (EMT), and apoptosis. Trastuzumab inhibits HER2 signaling and can engage in antibody-dependent cell-mediated cytotoxicity (ADCC). Pertuzumab can also mediate ADCC and inhibits HER2 dimer formation, abrogating HER2 intracellular signaling. The trastuzumab-based antibody–drug conjugate T-DM1 maintains the functions of trastuzumab but also delivers a cytotoxic payload (emtansine) on internalization of the receptor/T-DM1 complex. Lapatinib and neratinib are small-molecule tyrosine kinase inhibitors targeting the cytoplasmic ATPase domain of HER family members. This figure is adapted from publications by Roskoski et al. [15], Segovia-Mendoza et al. [24], Kourie et al. [25], Appert-Collin et al. [26] and references cited therein; further detail can be found within those publications. Signaling pathway interactions adapted from the KEGG pathway database. AKT, protein kinase B; EGF, epidermal growth factor; EGFR, epidermal growth factor receptor; ERK, extracellular signal-regulated kinase; HB-EGF, heparin-binding EGF-like growth factor; JAK, Janus kinase; MEK, mitogen-activated protein kinase kinase; NRG, neuregulin; PI3K, phosphoinositide 3-kinase; PKC, protein kinase C; PLCγ, phospholipase C-γ; PTEN, phosphatase and tensin homolog; STAT, signal transducer and activator of transcription; TGF, transforming growth factor.

Eleven epidermal growth factor (EGF)-like ligands have been described (Figure 1). These ligands can be receptor-specific, e.g., EGF, transforming growth factor-α, and epigen for EGFR, or non-specific, e.g., neuregulins for HER3 and HER4, and epiregulin for EGFR and HER4 [15,16]. HER2 has no known ligand. Each ligand comprises approximately 55 amino acids and shares a conserved EGF-like pattern of three disulfide bonds and a loop-rich structure [17,19,21]. Ten HER-family homo- or heterodimer combinations are possible; however, HER2 is the preferred dimerization partner as it exists in an open conformation similar to a ligand-activated state [15,22]. HER2-containing dimers have a lower dissociation rate and result in greater signal transduction compared with other dimers [23].

### 2.1. HER2 Overexpression

HER2 overexpression is found in 15–20% breast cancers [27]. For other malignancies, Yan et al. reported HER2 overexpression in 4.7%, 11.3%, 12.4%, and 9.8% of gastric, esophageal, bladder, and gallbladder cancers, respectively, and in 6.3% of cholangiocarcinomas [28]. The development of trastuzumab, the first clinically approved HER2-targeted agent, revolutionized outcomes for patients with HER2+ breast cancer [29,30]. Before the advent of trastuzumab, HER2+ breast cancer was considered the clinical subtype with the worst prognosis. Multiple HER2-targeting agents have since been developed, including antibody therapies such as pertuzumab, and small-molecule TKIs, such as lapatinib and neratinib, the only two TKIs approved for treatment of HER2+ breast cancer.

### 2.2. HER2 Mutation

Point mutations in genes for all four HER family members have been reported in a variety of cancers as reviewed in the COSMIC database (https://cancer.sanger.ac.uk/cosmic). Activating *HER2* mutations tend to occur in the absence of *HER2* amplification and their presence may represent a novel patient population potentially served by treatment with HER2-directed agents [6].

## 3. Lapatinib and Neratinib: HER-Targeting Small-Molecule TKIs

Small molecules, such as lapatinib and neratinib, have a number of inherently advantageous characteristics over monoclonal antibody therapies, including the ability to target multiple kinase family members simultaneously, to act directly at the site of intracellular signaling, and potentially to cross the blood–brain barrier [31]. In addition, as these TKIs bind intracellularly, they can inhibit p95HER2, the highly active, truncated form of HER2 that lacks most of the ECD, which trastuzumab and other ECD-directed antibody therapies cannot [2,32]. The similarities of and differences between lapatinib and neratinib are discussed below and summarized in Table 1.

### 3.1. Lapatinib: A Reversible TKI

Lapatinib (Tykerb^®^; Tyverb^®^; Novartis International AG, Basel, Switzerland) is a reversible TKI of EGFR and HER2. Lapatinib was approved by the FDA in 2007 and is indicated for use with capecitabine in patients with metastatic HER2+ breast cancer whose disease progressed on trastuzumab [8,33]. Lapatinib was approved in 2010 for use with letrozole in the treatment of patients with HER2+, estrogen receptor-positive (ER+) breast cancer [34]. Lapatinib was granted marketing authorization by the European Commission in 2008, and is indicated for use in combination with: capecitabine for patients with advanced or metastatic disease with progression following prior therapy, which must have included anthracyclines and taxanes, and therapy with trastuzumab in the metastatic setting; trastuzumab in patients with hormone receptor-negative metastatic breast cancer that progressed on prior trastuzumab in combination with chemotherapy; and an aromatase inhibitor for postmenopausal women with hormone receptor-positive metastatic disease, not currently intended for chemotherapy [35].

**Figure 2:**
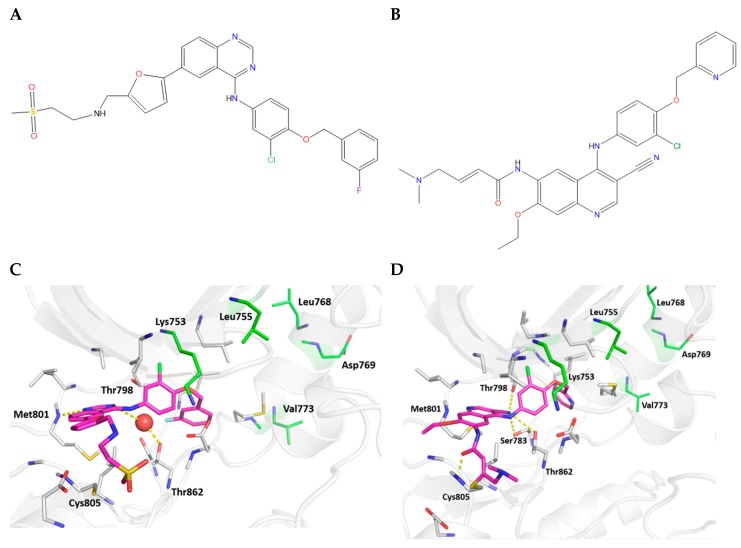
Chemical structures of (**A**) lapatinib and (**B**) neratinib. Modeled structures of the complexes between (**C**) HER2 and lapatinib and (**D**) HER2 and neratinib. The crystal structure of the HER2 kinase domain in its inactive form (PDB ID: 3PP0) was taken and lapatinib (guided by the crystal structure of the complex between lapatinib and HER4, PDB ID 3BBT) and separately neratinib (guided by the crystal structure of the complex of neratinib and EGFR, PDB ID 2JIV) were modeled into the binding pocket. Lapatinib and neratinib are shown as colored sticks, with carbon atoms in magenta, nitrogen atoms in blue, oxygen atoms in red, sulfur atoms in yellow, chlorine atoms in green, and fluorine atoms in cyan. Lapatinib is bound non-covalently, whereas neratinib is covalently attached to the sulfur of Cys805. All interacting residues are highlighted as thin sticks (gray carbon) and all interactions are highlighted as dotted lines (yellow). *HER2* mutations that are resistant to lapatinib are highlighted (green carbon, stick representation). A key water molecule (red sphere, Panel C) that mediates interactions between lapatinib and HER2 is shown. Hydrogen bonds made by the ligands are shown as thick yellow dashes. Lapatinib is engaged in hydrophobic interactions, one hydrogen bond with the backbone amide of Met801 and one water-mediated hydrogen bond with the side-chain hydroxyl of Thr862. Neratinib is engaged in hydrophobic interactions, forming a hydrogen bond with the side chain of Ser783, a hydrogen bond with the side chain of Thr862, a hydrogen bond with the backbone of Cys805, and a hydrogen bond with the side-chain hydroxyl of the gatekeeper Thr798. Modeling, docking, and optimization using molecular dynamics simulations (250 ns simulations of each complex performed in triplicate) of the complexes were performed using standard methods ([43]; in the current study we used Amber [44] version 16 and the Amber ff14SB force field [45]). HER, human epidermal growth factor receptor.

In 2001, Rusnak et al. described the activity of GW2016 (GW572016; lapatinib; Figure 2A) in preclinical models [36]. In vitro kinase assays revealed half maximal inhibitory concentration (IC_50_) values against purified EGFR and HER2 of 10.8 and 9.2 nM, respectively. Proliferation IC_50_ values were <160 nM in tumor cell line models of EGFR and HER2 overexpression, 100-fold less than observed with cell line models of normal tissue. Cytotoxicity as opposed to growth arrest was confirmed using outgrowth and bromodeoxyuridine-incorporation assays, along with propidium iodide staining in head and neck (HN5) and HER2+ breast cancer (BT474) cell line models. Lapatinib displayed concentration-dependent growth inhibition of tumor xenografts using these same cell line models in vivo [36]. When bound to HER2, lapatinib inhibits both PI3K/protein kinase B (AKT) and MAPK signaling pathways [37]. MAPK signaling inhibition leads to increased expression of BCL2 interacting mediator of cell death (BIM), which results in apoptosis. Lapatinib can also induce apoptosis via decreasing levels of survivin and increasing levels of myeloid cell leukemia-1 (MCL-1) [38,39,40,41]. Lapatinib also induces stabilization and accumulation of HER2 on the cell surface, which may allow for greater trastuzumab-induced antibody-dependent cell-mediated cytotoxicity (ADCC) (see Section 4.6 for more detail) [42].

### 3.2. Neratinib: An Irreversible TKI

Neratinib (Nerlynx^®^; Puma Biotechnology, Inc., Los Angeles, CA, USA) is an irreversible pan-HER TKI approved by the FDA in 2017 [9]. Neratinib is indicated for the extended adjuvant treatment of adult patients with early-stage HER2-overexpressed/amplified breast cancer, following adjuvant trastuzumab-based therapy [14]. Neratinib was granted marketing authorization by the European Commission in 2018 and is indicated for extended adjuvant treatment of adult patients with early-stage hormone receptor-positive HER2-overexpressed/amplified breast cancer who are less than 1 year from the completion of prior adjuvant trastuzumab-based therapy [46].

Neratinib (compound 25o, HKI-272), an anilinoquinoline derivative of pelitinib (EKB-569; Wyeth; Figure 2B) [46], was discovered during screening of 6,7-disubstituted-4-(arylamino)quinoline-3-carbonitrile derivatives [47], and was one of six novel compounds deemed to be the best candidate dual inhibitors of HER2 and EGFR, as assessed by kinase and cell-proliferation assays. Subsequent to extensive toxicological and pharmacokinetic studies [47,48], neratinib was chosen for clinical evaluation.

Kinase assays revealed that neratinib inhibited HER2 and EGFR activity at nanomolar concentrations (mean IC_50_ ± standard error [SE]: 59 ± 13 nM and 92 ± 17 nM, respectively), without significantly affecting the activity of a panel of serine/threonine kinases, including AKT, cyclin D1/ CDK4, cyclin E/CDK2, cyclin B1/CDK1 and c-RAF, or the tyrosine kinase c-Met [48]. Neratinib has been reported to inhibit HER4 activity at a mean IC_50_ of 19 nM [48]. Protein-binding dye proliferation assays in a panel of cell lines with varying EGFR and HER2 expression confirmed the selectivity of neratinib for these receptors with low nanomolar IC_50_ values (2–3 nM) for HER2-overexpressing cell lines (SKBR3, BT474, and 3T3/neu) and a slightly higher IC_50_ value for the EGFR-overexpressing epidermoid carcinoma cell line A431 (mean ± SE: 81 ± 9 nM) [48]. Mean ± SE IC_50_ values in HER2/EGFR-negative cell lines (3T3, MDA-MB-435, and SW620) were all ≥690 ± 84 nM. Neratinib inhibited ligand-dependent and -independent phosphorylated HER2 and EGFR activity and downstream MAPK and AKT signaling (Figure 1) [48]. Neratinib, which was confirmed to bind covalently based on its antiproliferative effect in a cell line model despite withdrawal of the drug, was found to have a concentration-dependent impact on cell cycle-related cyclin D1, protein 27, and retinoblastoma protein. Finally, neratinib significantly inhibited tumor growth in vivo in xenograft models overexpressing HER2 (3T3/neu and BT474) and EGFR (SKOV-3 and A431) [48]. The pharmacodynamics, pharmacokinetics, and potential therapeutic niches of neratinib have been reviewed [25,49,50].

## 4. Differentiating Features of Lapatinib and Neratinib

### 4.1. Impact on HER-Family Dimerization

As well as inhibiting intracellular phosphorylation, lapatinib and neratinib have been shown to interfere with HER receptor dimerization, unlike other TKIs such as gefitinib and erlotinib [51]. Because of their structure and binding characteristics, lapatinib and neratinib bind to the inactive conformation of HER family members, restricting ligand-induced activation (Figure 2C,D) [7]. However, Claus et al. recently provided evidence from two- and three-dimensional modeling of HER2+ breast cancer that lapatinib and neuregulin synergize to enhance proliferation [52]. The authors suggested that lapatinib induces a HER2/HER3 conformation that is distinct from the canonical ligand-induced heterodimer, is dependent on the ability of HER3 to bind adenosine 5′-triphosphate (ATP), and is primed for activation by neuregulin. Interestingly, this synergistic effect was not observed with neratinib and neuregulin in the same model. The authors postulated that the irreversible, covalent nature of neratinib binding, as opposed to the reversible, non-covalent lapatinib binding, explains this difference. A later study by Canonici et al. provided similar results and is covered in more detail in Section 4.4 [53].

### 4.2. Characterization of the Kinomes Inhibited by Lapatinib and Neratinib

Small-molecule kinase inhibitors are designed to target the functionally and structurally conserved ATP binding site of kinases. This frequently leads to polypharmacology, or off-target sites of action. Type II kinase inhibitors, including neratinib and lapatinib, bind kinase domains in a DFG (Asp-Phe-Gly)-out, inactive kinase conformation, which allows them access to an allosteric binding pocket adjacent to the ATP site, conferring greater selectivity in most, but not all, cases. Type I kinase inhibitors generally bind kinases at the ATP site in their active conformation, and therefore do not have access to the allosteric binding pocket, resulting in greater selective variability [54].

**Figure 3:**
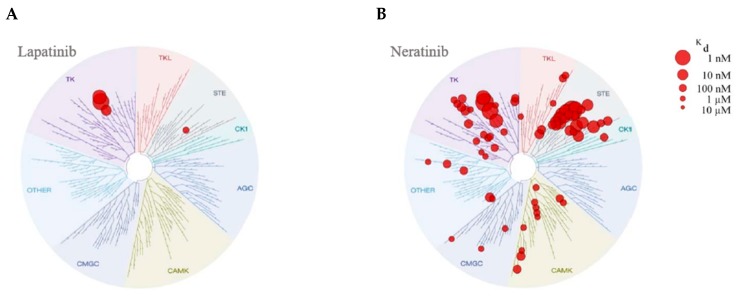
KINOME*scans*^®^ (Eurofins DiscoveRx, San Diego, CA, USA) of (**A**) lapatinib and (**B**) neratinib. Each red circle indicates a kinase found to bind to the drug. Larger circles indicate higher affinity interactions. Interactions with K_d_ < 3 μM are shown. Reprinted by permission from Springer Nature: Nature Biotechnology (Comprehensive analysis of kinase inhibitor selectivity, Davis MI, et al., copyright 2011 [7]). K_d_, equilibrium dissociation constant.

Kinome inhibition experiments have demonstrated significantly different binding patterns for neratinib and lapatinib. Davis et al. screened the activity of a set of 72 known kinase inhibitors, including neratinib and lapatinib, against a panel of 442 kinase competition-binding assays representing >80% of catalytically active human protein kinase domains [7]. Lapatinib, which is not a typical Type II inhibitor because of its unusual displacement of the α-C helix [55], was among the most selective of the inhibitors tested (Figure 3A). Neratinib was less selective than lapatinib, targeting EGFR, HER2, and HER4, along with members of the TK and STE kinome subdomains, particularly KHS1/KHS2 (Figure 3B). Target kinases with lower binding affinities were also identified in the TKL, CMGC, AGC, CK1, and CAMK subdomains. Klaeger et al. used a chemical proteomic approach (kinobeads) and quantitative mass spectrometry to analyze the target kinome of 243 clinically relevant kinase inhibitors, including lapatinib and neratinib [56]. Their findings confirmed the results of the study by Davis et al. [7], with lapatinib proving more selective than neratinib. The Klaeger et al. data are accessible through proteomicsDB (https://www.proteomicsdb.org/#projects/4257); the Davis et al. database can be visualized through KinMap (http://kinhub.org/index.html) using the Profiling function.

High selectivity in a kinase inhibitor is usually advantageous for cancer subtypes driven by a particular target, as reduced off-target effects generally lead to reduced cytotoxicity; however, targeting a larger proportion of the kinome also has its advantages. Neratinib’s more complete irreversible inhibition of the disease-related HER family is thought to prevent the development of resistance mediated through other HER family members [57]. Lapatinib has high affinity for EGFR and HER2 but is reversible, which leads to incomplete inhibition and reactivation of its targets [58]. Neratinib also binds with high affinity to MEK1 and MEK2, targets downstream of the HER family, potentially adding to its antiproliferative effects [59].

The off-target effects of neratinib may contribute to improved synergy with other targeted therapies and chemotherapies, such as dasatinib, which also inhibit kinases in the TK, TKL, and STE subdomains [60]. It should be noted, however, that although neratinib is a less selective inhibitor than lapatinib as visualized through inhibition of the kinome, it does not bind irreversibly off-target in the absence of the requisite cysteine residues. This distinction between affinity for its primary targets and the targets to which neratinib covalently binds allows for different administration and half-lives of activity for on- versus off-target effects. In addition, the measured binding affinities for neratinib kinome targets of 100–3000 nM suggest that many of the interactions may not have clinical significance as the intra-tumoral concentrations required may not be achieved with approved doses in patients (240 mg; mean ± standard deviation maximum plasma concentration 75.9 ± 12.9 ng/mL; 113 ± 19 nM) [61].

### 4.3. Mechanistic Interaction with the HER Family

In addition to differences in their selectivity profiles, lapatinib and neratinib also vary in how they interact with HER family members. Using existing crystal structure data for EGFR–erlotinib interactions as a template, Tsou et al. built a homology model of the HER2 catalytic domain [47]. The authors proposed a mechanism for inhibition whereby the headpiece of the neratinib molecule inserts into the ATP binding pocket, allowing an electron-withdrawing group in close proximity to a double bond (a Michael acceptor) to be positioned so as to interact with a cysteine residue of EGFR that is analogous to cysteine residues in other HER family members. This arrangement leads to a covalent interaction between the β-carbon and the cysteine residues [47]. The irreversible covalent binding of neratinib to Cys-797 of EGFR overcomes resistance to the gatekeeper *EGFR* T790M mutation, a common resistance mechanism to erlotinib or gefitinib in L858R-mutated lung cancer [62,63].

### 4.4. Cytotoxicity in HER2+ and EGFR+ Cell Lines

The cytotoxicities of lapatinib and neratinib have been widely investigated. Both agents have demonstrated in vitro and in vivo efficacy against HER2- and EGFR-amplified breast cancer cell lines, with neratinib demonstrating significantly lower IC_50_ values in cell line models. Neratinib was more potent than lapatinib in each cell line examined and this was the case for both ER+/HER2+ and ER-negative/HER2+ cell lines (Table 2). A caveat to IC_50_ comparisons is that many studies were performed under different conditions, using different assays to assess proliferation. Nonetheless, some notable differences between the agents have been observed.

Canonici et al. examined sensitivity to neratinib in a panel of 36 breast cancer cell lines including 12 HER2+ cell lines [73]. All HER2+ cell lines examined were more sensitive to neratinib than lapatinib based on previously published IC_50_ values for lapatinib in the same cell lines [67]. Neratinib sensitivity correlated with total and phosphorylated HER2 levels in the HER2-positive breast cancer panel. Similarly, Konecny et al. showed that total HER2 levels, and not EGFR levels, were predictive of in vitro response [37]. In a panel of trastuzumab-sensitive cell lines, trastuzumab plus neratinib was more potent than either drug alone [73]. In contrast, in a panel of trastuzumab-resistant HER2+ cell lines sensitive to neratinib, addition of trastuzumab did not provide added benefit.

Neratinib was compared with lapatinib and with afatinib, another irreversible pan-HER TKI, in a panel of 11 HER2+ breast cancer cell lines [53]. Neratinib was more potent than lapatinib in all 11 cell lines in this study and was more potent than afatinib in nine. Each of the cell lines was classified as sensitive or resistant to each of the TKIs: three displayed innate resistance to all three TKIs (JIMT-1, MDA-MB-453, and UACC-732). Combination proliferation assays were carried out in three of the HER2+ breast cancer cell lines that were: (1) sensitive to trastuzumab/lapatinib (SKBR3); (2) resistant to the combination of trastuzumab/lapatinib/neratinib/afatinib (MDA-MB-453); and (3) sensitive to lapatinib/resistant to trastuzumab (HCC1569). Addition of any of the three TKIs improved response to trastuzumab in SKBR3, but addition of pertuzumab did not. Combination with a TKI did not enhance response to trastuzumab in the MDA-MB-453 or HCC1569 cell lines but the triplet of trastuzumab/pertuzumab/neratinib showed the strongest antiproliferative effect. Addition of the ligands amphiregulin and heregulin altered responses; in the presence of either ligand, increasing the concentration of trastuzumab, pertuzumab, or neratinib alone had no effect, but growth inhibition was significant when all three inhibitors were combined.

A comprehensive study by Stanley et al. examined a panel of breast cancer cell lines for HER-family expression and sensitivity to reversible and irreversible HER-family inhibitors alone or in combination with other targeted kinases and chemotherapeutic agents [57]. In this panel, which included the SKBR3, BT474, and MDA-MB-453 HER2+ cell lines and the MDA-MB-468 EGFR+ cell line, IC_50_ values were lower for neratinib than lapatinib for all cell lines examined. Neratinib IC_50_ values were equivalent to or lower than those of afatinib. The authors found no statistically significant relationship between EGFR, HER2, or HER3 expression and response to lapatinib or neratinib. Analysis of the phosphorylation of HER family members in the SKBR3 cell line, with or without the HER-family ligands EGF, heparin-binding EGF, and neuregulin, found that irreversible HER-family TKIs were more effective than reversible TKIs at inhibiting phosphorylation of HER-family receptors and downstream signaling molecules (AKT/MAPK).

In summary, preclinical assessment of cytotoxicity suggests several differences between neratinib and lapatinib. Neratinib is a more potent inhibitor of proliferation and downstream signaling pathways than lapatinib or afatinib in cell line models of HER2+ breast cancer and can potentiate the effects of trastuzumab in trastuzumab-sensitive HER2+ breast cancer cell lines. Cell line models with innate or acquired trastuzumab resistance are sensitive to neratinib, whereas neratinib-resistant cell line models are cross-resistant to trastuzumab, lapatinib, and afatinib. Incomplete inhibition of HER family members by lapatinib may be a determining factor that allows resistance to develop through activation of EGFR/HER3 and HER4. The addition of a TKI to trastuzumab is beneficial, with both lapatinib and neratinib improving response to trastuzumab in trastuzumab-sensitive HER2+ cells; the addition of pertuzumab does not enhance this effect. The triple combination of trastuzumab, pertuzumab, and neratinib was more effective than the lapatinib triplet in innately HER2-targeted therapy-resistant cell line models.

### 4.5. Cytotoxicity in HER2-Mutant Cell Lines

A recently identified patient population that may benefit from HER2 inhibitors are those whose tumors harbor activating *HER2* mutations, even in the absence of gene amplification. Multiple studies have reported that oncogenic *HER2* mutations occur primarily in the kinase domain of HER2 [74,75]. Analysis of eight breast cancer genome-sequencing studies identified 13 *HER2* mutations from 25 patients with non-*HER2*-amplified breast cancer (Table 3). Functional characterization classified seven of the 13 mutations as HER2 activating, based on increased EGFR and HER3 phosphorylation compared with wild-type *HER2* [75]. In mouse xenograft studies, NIH3T3 cells transduced with three mutations (V777L, D769H and G309A) displayed more rapid tumor growth than *HER2* wild-type controls [75].

An understanding of which *HER2* mutations are responsive to which HER2 inhibitors is critical. For example, the *HER2* L755S mutation is known to be associated with lapatinib resistance. When retrovirally induced in the MCF-10A non-transformed breast epithelial cell line, *HER2* L755S mediated resistance to lapatinib—but not neratinib—as determined by inhibition of both cell growth and HER2 phosphorylation [75]. A further study of *HER2* L755S reported that this mutation arose independently in two resistant cell line models derived from BT474: the lapatinib-resistant BT-474-AZ/LR and the lapatinib- and trastuzumab-resistant BT474/ATCC-LTR cell lines [72]. Small interfering RNA knockdown of *HER2* L755S reversed this resistance, whereas ectopic expression of *HER2* L755S conferred resistance to lapatinib in treatment-naive BT474, SKBR3, and AU565 cell lines. Neratinib also overcame resistance to lapatinib and lapatinib/trastuzumab in two BT474 cell line models harboring the L755S mutations [72]. Further, the non-small cell lung cancer-associated *HER2* L755P mutation also mediated lapatinib resistance [77]. Neratinib was more potent by orders of magnitude than lapatinib in six of the *HER2*-mutant MCF10A and *HER2* wild-type-transduced MCF10A cell lines [75]. In 3-dimensional Matrigel^®^ (BD Biosciences, San Jose, CA, USA) assays, the *HER2* mutation P780_Y781insGSP conferred resistance to trastuzumab and lapatinib, but not to neratinib. A patient with *HER2* L869R-mutant breast cancer initially achieved a partial response following neratinib treatment, followed by disease progression with acquisition of a secondary ‘gatekeeper’ *HER2* mutation, T798I [78]. In vitro analysis showed that although neratinib was ineffective against this dual *HER2* mutant, afatinib and the osimertinib metabolite AZ5104 could inhibit HER2 phosphorylation.

Others have suggested that cancers harboring exon 20 mutations that mediate resistance to lapatinib/trastuzumab may be sensitive to neratinib treatment [79]. Koga et al. reported lower sensitivity index values for neratinib than lapatinib in lung adenocarcinomas harboring the *HER2* exon 20 insertion mutations A775_G776insYVMA, G776delinsVC, and P780_Y781insGSP [80]. In the SUMMIT trial, 50% of patients in the breast cancer cohort in whom exon 20 insertions were observed responded to treatment with neratinib, unlike the non-small cell lung cancer cohort, in which response to neratinib was limited in patients with such mutations [81].

Co-occurrence of *HER2* amplification and mutation has also been documented. Screening of 22 *HER2* exons in 1248 primary breast cancers and 18 matched metastatic samples identified *HER2* mutation in 2.24% of tumors (28 of 1248) [76]. In that study, mutation rates were similar in HER2+ and HER2-negative tumors: among HER2+ tumors, the *HER2* mutation rate was 2.31% (21 of 910) versus 2.07% (7 of 338) in HER2-negative tumors. Functional analysis of these mutations suggested that L755S and K753E mutations were associated with resistance to lapatinib and trastuzumab but not neratinib. In NIH3T3 cells, those bearing the *HER2* L768S and V773L mutations formed tumors more rapidly than those with wild-type *HER2*. MCF10A, BT474, and MDA-MB-231 cells bearing the K753E mutation were resistant to lapatinib but not neratinib, and the drug-resistant *HER2* K753E and L755S mutations were enriched in metastatic lesions [76].

In their study of breast tumors with amplified and mutated *HER2*, Cocco et al. showed that coincident *HER2* mutation and amplification was associated with poor response to trastuzumab and lapatinib in cultured cell lines [5]. In mice, xenografts established from a patient with a HER2+ tumor with an acquired D769Y mutation in *HER2* following progression on trastuzumab-based therapy were resistant to trastuzumab and lapatinib but sensitive to neratinib, which induced durable tumor shrinkage.

Clinically, the therapeutic relevance of targeting HER family mutations is exemplified by SUMMIT (NCT01953926), an ongoing, multicenter, multi-histology, phase II precision medicine ‘basket’ trial investigating the efficacy and safety of neratinib in patients with *HER2*-mutant cancers [81]. Interestingly, neratinib efficacy depended on both the specific mutation and on tumor histology, with breast, cervical, and biliary cancers showing the greatest response [81]. Given the relative scarcity of *HER2* mutations, the observational genomic-screening protocol HER-Seq (NCT03786107) was designed to identify patients potentially eligible to enroll into neratinib treatment protocols such as SUMMIT, by performing *HER2*-targeted next-generation sequencing on plasma collected from patients with metastatic breast or cervical cancer.

### 4.6. Downstream Signaling or Gene Expression

Differences in the characteristics of lapatinib and neratinib, as outlined above, have been shown to affect downstream signaling pathways, resulting in differential responses to each drug. The expression levels of five genes (*RB1CC1*, *FOXO3a*, *NR3C1*, *ERBB3* and *CCND1* [cyclin D1]) were altered by lapatinib treatment, in proportion to lapatinib sensitivity [68]. A follow-up study examined the expression of these genes in response to lapatinib, neratinib, and afatinib in three HER2+ cell lines (TKI-sensitive SKBR3 and BT474, and lapatinib-insensitive MDA-MB-453) using TaqMan^TM^ real-time polymerase chain reaction [69]. Lapatinib and neratinib were reported to have a similar impact pattern on the gene panel across all three cell lines, although the magnitude of change in four of the five genes was greater in neratinib- versus lapatinib-treated SKBR3 and BT474 cells. *CCND1* levels have also been reported to be altered following neratinib treatment [48].

A quantitative, label-free, liquid chromatography-mass spectrometry proteomic approach was used to investigate cell line models of HER2+ breast cancer following short-term treatment with TKIs [82]. Twelve-hour treatment with lapatinib, neratinib, and afatinib was examined in the BT474 cell line. Twenty-one proteins were altered significantly in response to neratinib (150 nM), whereas 16 proteins were altered significantly in response to lapatinib (1 µM). Within this dataset, six proteins had altered levels in response to short-term exposure to both lapatinib and neratinib but not afatinib (lamina-associated polypeptide 2 isoform α, DNA-dependent protein kinase catalytic subunit, drebrin, myristoylated alanine-rich C-kinase substrate, 78 kDa glucose-regulated protein, and poly[ADP-ribose] polymerase 1). Three proteins were co-altered by afatinib and neratinib but not lapatinib (heterogeneous nuclear ribonucleoprotein H, interleukin enhancer-binding factor 3, and trifunctional enzyme subunit beta, mitochondrial), which are involved in mRNA splicing, apoptosis/protein metabolism, and protein acetylation, respectively. Finally, only two proteins were co-altered in response to all three inhibitors (trifunctional enzyme subunit α and heterogeneous nuclear riboprotein R). The breadth of function of these proteins indicates the potential implications that inter-TKI differences could have in tumor cell responses to these therapies. The protein products of the genes examined as part of the five-gene panel [69] were not apparent in the proteomics study, which may be related to temporal differences between RNA and protein expression in response to drug exposure.

### 4.7. HER2 Receptor Levels and Endocytosis

Cell surface levels of RTKs are regulated by endocytosis, a process through which RTKs are internalized into endosomes, where they can be recycled back to the cell surface or progress to lysosomes for degradation; this process is accelerated by ligand binding [83]. EGFR homodimerization and EGFR/HER2 heterodimerization occur with comparable affinities, and EGFR/HER2 complexes internalize as a single entity [84]. Studies of HER2 internalization have revealed a dependency on the chaperone heat-shock protein (HSP)90 for stability at the cell surface [85,86]. HSP90 inhibitors can induce HER2 degradation through proteasomal or lysosomal pathways, which are regulated by ubiquitylation [87,88,89]. NVP-AUY922, an HSP90 inhibitor, has been reported to inhibit growth of HER2+ and trastuzumab-resistant breast cancer cells [90]. In vitro, NVP-AUY922 enhanced response to trastuzumab but not to chemotherapy. Nuclear localization of ligand-activated HER3 in proliferating cells has been associated with a clathrin-independent endocytic mechanism [91]. The internalization and lysosome targeting of HER2, HER3, and HER4 are reported to be less efficient than those of EGFR, suggesting that EGFR regulation could be affected by overexpression of other family members [86].

**Figure 4:**
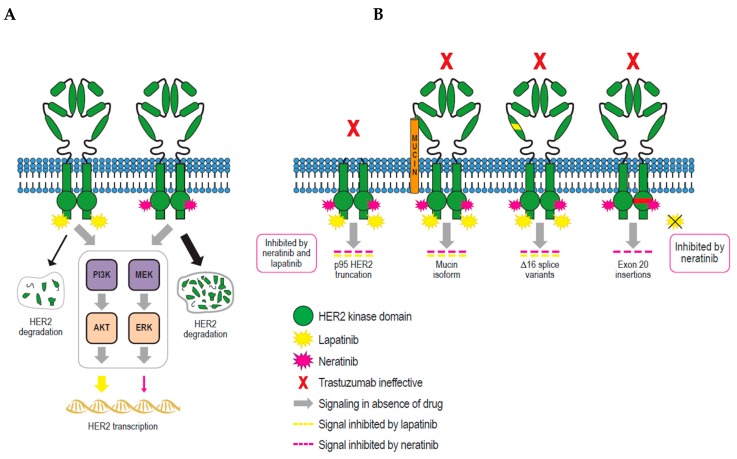
(**A**) Schematic representation of HER2 regulation by lapatinib and neratinib via elevation of *HER2* transcription and endocytosis as proposed by Zhang et al. [59]. The differential effects of neratinib and lapatinib are indicated by weighted arrows, with thicker arrows corresponding to a greater effect. Both lapatinib and neratinib increase *HER2* transcription through inhibition of PI3K and MAPK signaling, but lapatinib to a greater extent. Both tyrosine kinase inhibitors increase endocytosis and lysosomal degradation of HER2 but neratinib leads to greater HER2 ubiquitylation and degradation than lapatinib [59]. The net effect is an increase in cellular HER2 levels with lapatinib exposure and a decrease in cellular HER2 levels following exposure to neratinib. (**B**) Schematic depiction of HER2-related mechanisms implicated in resistance to trastuzumab, lapatinib, and neratinib. Adapted from Arteaga and Engelmen [79] and references cited therein. Trastuzumab (or pertuzumab/T-DM1) cannot bind truncated p95 HER2. Expression of specific mucin isoforms can abrogate trastuzumab binding. The Δ16 HER2 splice variant, which has an in-frame deletion of 16 amino acids (634–649) of exon 16 in domain IV, does not bind trastuzumab. The Δ16 HER2 splice variant co-exists with wild-type HER2 and can promote transformation through constitutively active homodimer formation and enhanced signaling activity [93]. All three mechanisms mediate resistance to trastuzumab but are susceptible to lapatinib and neratinib inhibition. The P780_Y781insGSP exon 20 insertion in the HER2 kinase domain has been shown to mediate resistance to lapatinib and trastuzumab, but not neratinib, in a breast cancer cell line model [75]. AKT, protein kinase B; ERK, extracellular signal-regulated kinase; HER, human epidermal growth factor receptor; MEK, mitogen-activated protein kinase kinase; PI3K, phosphoinositide 3-kinase; T-DM1, trastuzumab emtansine.

Evidence has accumulated that lapatinib and neratinib have differential effects on cell surface levels of HER2, with lapatinib increasing and neratinib decreasing levels [42,92]. Zhang et al. provided a hypothesis for the underlying mechanism responsible for these effects. In three HER2+ cell lines (SKBR3, AU565, and HCC1954), neratinib augmented endocytic degradation of HER2 through increased ubiquitylation, with only moderate increases in *HER2* transcription, whereas lapatinib led to strong upregulation of *HER2* transcription and more limited HER2 endocytosis (Figure 4A) [59].

The impact of TKI-altered HER2 levels on ADCC response to trastuzumab has been investigated as a potential rationale for their combination. Lapatinib-induced increases in HER2 resulted in increased trastuzumab-mediated ADCC in HER2+ breast, gastric, and mesothelioma cell line models [42,92,94,95,96]. However, the impact of lapatinib and neratinib on ADCC may be dependent on factors other than HER2 antigen levels, as both TKIs led to increases in ADCC in vitro, dependent on the cytotoxic capacity of immune effector cells involved [92]. Profiling of lapatinib- and neratinib-resistant HER2+ breast cancer cell lines showed that a TKI resistance phenotype in vitro is associated with reduced trastuzumab-related ADCC, which can also be independent of HER2 expression levels [97,98].

The ECD of HER2 can be cleaved by matrix metalloproteinases to release a soluble form of HER2 (sHER2) into the peripheral circulation [99]. A high baseline level of circulating sHER2 is prognostic for shorter disease-free survival in early-stage, resected HER2+ breast cancer; high sHER2 levels at recurrence also predict shorter survival [100]. Trastuzumab has been shown to inhibit sHER2 shedding by inhibiting basal and activated proteolytic cleavage of HER2 [101]. Conversely, lapatinib increases inactive HER2 levels at the tumor cell surface and augments release of sHER2 [102]. Higher sHER2 levels predict improved progression-free survival with lapatinib treatment independent of tumor levels of HER2 in patients with advanced breast cancer [103]. Similar data are not currently available for neratinib.

## 5. Resistance to Lapatinib and Neratinib

Despite the clinical successes seen with HER2-targeted therapies, development of resistance is an ongoing problem. In vitro cell line models have been used extensively to examine possible mechanisms of resistance to lapatinib and neratinib. This involves either continuous exposure to the TKI, dose-escalation treatment, or single cell cloning. Section 4.5 details mutations associated with TKI resistance. Some possible resistance mechanisms are shown in Figure 4B.

### 5.1. Lapatinib Resistance

Many potential lapatinib resistance mechanisms have been proposed. These can be categorized broadly as: activation of alternative tensin homolog RTKs; downstream reactivation of PI3K or MAPK signaling; or phenotypic switching.

Although proliferation of HER2+ breast cancer cells is driven by HER2 signaling, redundant survival mechanisms can be activated when HER2 signaling is suppressed. These include upregulation of other RTKs, such as alternative HER family members, MET, fibroblast growth factor receptor-2, and insulin-like growth factor-1 receptor [104,105,106]. In one study using four cell lines with acquired lapatinib resistance, lapatinib inhibited HER2 activity but downstream PI3K signaling persisted, despite an absence of *PIK3CA* mutation or phosphatase and tensin homolog (PTEN) loss [107]. Resistance was mediated by EGFR–HER3 dimerization, which was regulated by increased levels of membrane-bound heregulin. Interestingly, neratinib overcame this lapatinib resistance. HER4 signaling may also be a mechanism of resistance: Canfield et al. showed that HER2+ breast cancer cell line models of lapatinib and trastuzumab resistance (BT474-LR, SKBR3-TR, SKBR3-LR, and SKBR3-LTR) were dependent on HER4 expression for survival, unlike their parent cell lines [108]. The authors suggested that the efficacy of several pan-HER family inhibitors (afatinib, canertinib, dacomitinib, varlitinib, and neratinib) in lapatinib-resistant HER2+ breast cancer models was potentially dependent on their ability to inhibit HER4 activity. The authors also reported the findings of an in vivo study, which showed membranous tumor HER4 expression in all lapatinib-treated HER2+ tumors [108].

Alternatively, lapatinib resistance may occur through downstream reactivation of PI3K, which may be due to activation by Src family kinases, through insulin receptor substrate 4, an indirect cytoplasmic activator of PIK3CA, or by the acquisition of *PIK3CA* or *HER2* mutations [109,110,111].

ER pathway activation may also cause HER2-targeted therapy resistance. Li et al. showed that, despite continued inhibition of HER2 and PIK3CA, BT474 cells with acquired lapatinib resistance proliferated in the presence of 5 µM lapatinib as a result of increased ER and MAPK signaling, which could be overcome by addition of fulvestrant [112]. Protein phosphatase 2A (PP2A) may also confer lapatinib resistance in vitro: increased PP2A activity was demonstrated in two cell lines with acquired lapatinib resistance, with PP2A inhibition leading to lapatinib resensitization [113].

Epithelial–mesenchymal transition (EMT) results in a stem-like phenotype associated with resistance to anti-cancer therapy [114,115]. Preclinical studies have implicated EMT in the emergence of HER2-targeted therapy resistance [116]. Lapatinib-resistant SKBR3 cells displayed EMT markers as well as reduced HER2 expression, and an epithelial-like phenotype could be re-established using the anti-integrin inhibitory antibody AIIB2 [117].

### 5.2. Neratinib Resistance

Despite its relatively recent approval for treatment of HER2+ breast cancer, several mechanisms of resistance to neratinib have been proposed, including decreased pro-apoptotic BCL2 family member expression, and increased cytochrome P450 (CYP) 3A4 activity [71,118]. Using neratinib-resistant HER2+ cell lines derived from SKBR3, ZR75-30, and BT474, Karakas et al. reported increased levels of anti-apoptotic MCL-1 and decreased levels of pro-apoptotic BCL2 family members BIM and p53 upregulated modulator of apoptosis (PUMA) in neratinib-resistant SKBR3 and ZR75-30 cells [118]. Neratinib-resistant BT474 cells also exhibited downregulated BIM and PUMA and increased BCL2 and BCL-XL levels in order to overcome neratinib-induced apoptosis [118]. Furthermore, these cell lines showed continued downstream activation of ERK-1/2, despite neratinib treatment. These neratinib-resistant cell lines were examined for sensitivity to ERK inhibition using the specific ERK1/2 inhibitor SCH772984, and sensitivity to BCL2/BCL-XL inhibition using the pan-BCL2 inhibitor ABT-737. Although the combination of neratinib and SCH772984 induced apoptosis in SKBR3-NR and ZR75-30-NR, the triple combination of neratinib, SCH772984, and ABT-737 was required to overcome neratinib resistance in BT474-NR cells [118].

Neratinib is also a substrate for CYP3A4, and increased CYP3A4 activity has been shown to cause neratinib resistance in cell line models. Breslin et al. developed HCC1954-NR and EFM192A-NR cell lines through continuous exposure to neratinib in vitro. These cell lines were not only neratinib resistant, but also cross-resistant to lapatinib and afatinib, more migratory and invasive than their parent cells, and displayed decreased HER2 expression [71].

The significance of PI3K pathway activation as a mechanism of resistance to neratinib has been inconsistent, appearing to depend on tumor type and disease setting. Preclinically, neratinib inhibited proliferation in *HER2*-amplified, *PIK3CA*-mutant tumor cell lines [73], and inhibited tumor growth in a HER2-positive, *PIK3CA*-mutant patient-derived xenograft model [119]. In the positive phase III ExteNET study, which compared 1 year of neratinib versus placebo given in the adjuvant setting after standard trastuzumab-based therapy, absolute risk reduction was associated with neratinib treatment of patients with *PIK3CA*-mutated or -amplified cancers, although this reduction was not statistically significant [120]. In the first 125 patients from the SUMMIT basket trial for *HER2*-mutant, metastatic solid tumors who were treated with neratinib monotherapy, *PIK3CA* mutations were more prevalent in patients who were on treatment for <24 weeks than those who were on for ≥24 weeks, although this difference was not statistically significant [81]. Larger clinical trials in specific tumor types may be required to determine the effect of *PIK3CA* mutation on response to neratinib in HER2-driven cancer.

In a recent study, Sudhan et al. demonstrated that the neratinib-resistant 5637 and OVCAR8 cell lines with activating *HER2* mutations, which had increased S6 kinase activity and S6 phosphorylation compared with neratinib-sensitive parental cells, were cross-resistant to lapatinib and afatinib [121]. S6 activation was primarily mediated by mammalian target of rapamycin (mTOR) complex 1 (mTORC1) pathway activation, which was attributed at least in part to RAS pathway upregulation; the combination of neratinib and everolimus overcame neratinib resistance in vitro and in vivo. Furthermore, patients with mTOR-activating alterations co-occurring with *HER2* mutations responded poorly to neratinib in the SUMMIT study [81]. The authors concluded that mTOR pathway alterations leading to reactivation of the HER2 signaling axis are important drivers of neratinib resistance in histologically distinct types of *HER2*-mutant cancers and that the combination of neratinib and mTORC1 inhibitors may be of clinical interest in patients with *HER2*-mutant cancers with mTOR pathway co-mutations [121]. Somatic *HER2* mutations may also have a role to play in neratinib resistance, with acquisition of a T798I gatekeeper mutation recently being reported in a neratinib-treated patient with ER+/*HER2*-mutant (L869R) breast cancer [78].

Other second-site (“on-target”) *HER2* mutations and amplifications have been detected in a subset of patients with *HER2* mutation-positive cancers progressing on treatment with neratinib in the SUMMIT trial. This suggests that hyperactivation of HER kinase signaling beyond a threshold of effective neratinib inhibition may be conferring both de novo and acquired resistance to therapy [122].

## 6. Novel Targeted Therapy Combinations

As suggested in Section 4.2, differences in the properties of lapatinib and neratinib are likely to impact efficacy when combined with other targeted agents. Histone deacetylases (HDACs) and histone acetyltransferases (HATs) play key roles in the epigenetic regulation of gene expression. HDAC inhibitors have multiple effects on cancer cells: cell cycle arrest, differentiation, and cell death; reduction of angiogenesis; and impact on the immune response [123]. The combination of lapatinib and entinostat has exhibited synergy in two in vivo models of HER2+ breast cancer (BT474 and SUM190), and entinostat-resensitized trastuzumab/lapatinib-resistant cell lines to lapatinib through induction of BIM1 [124]. The cytotoxicity of neratinib can be enhanced by HDAC inhibitors, with the added benefit that this combination has been shown to improve the anti-tumor immune response in preclinical models of afatinib-resistant non-small cell lung and breast cancers [125]. Addition of the cyclin-dependent kinase (CDK)4/6 inhibitor palbociclib to neratinib plus the HDAC inhibitor valproate suppressed growth of a patient-derived platinum/taxane-resistant ovarian xenograft model [126]. Neratinib plus valproate also enhanced the efficacy of a programmed cell death-1 (PD-1) antibody in a mouse syngeneic breast cancer model [125].

P-glycoprotein (P-gp; multidrug resistance protein 1 [MDR1]/ATP-binding cassette sub-family B member 1 [ABCB1]) is a drug efflux pump with a physiological role in the pharmacokinetics and pharmacodynamics of drugs, and it is a major mediator of tumor multidrug resistance [127]. Lapatinib and neratinib have been shown to function as P-gp inhibitors capable of reversing multidrug resistance in vitro [10,11,128]. Lapatinib has been shown to increase P-gp in cancer cell line models, while neratinib has been shown to do the opposite [71,129]. Further work is required to determine if these effects are dependent on the cell line lineage. The inhibition of P-gp by both TKIs could be considered a secondary anti-tumor function and combining lapatinib and neratinib with P-gp substrate chemotherapeutics may have value in P-gp overexpressing tumors.

The ER is known to play a role in resistance to HER2-targeted therapies as a compensatory signaling pathway [130,131]. Addition of anti-estrogens to lapatinib and neratinib has been shown to be of value in preclinical models [132,133]. The more complete inhibition of HER family signaling by neratinib versus lapatinib (Table 1) may further eliminate the HER family as a bypass pathway in the presence of ER inhibition, increasing the efficacy of anti-estrogens. The combination of neratinib and anti-estrogens is also synergistic in HER2-mutant models [134].

In addition, following ligand-induced proteolytic cleavage, the intracellular domain of HER4 (4ICD) has been shown to act as a co-activator of ER in the nucleus of ER+/HER2-low cells [135,136]. Unlike lapatinib, the ability of neratinib to potently inhibit HER4 may negatively impact the transcriptional co-factor activity of 4ICD. Neratinib plus fulvestrant is being examined in the ER+/*HER2*-mutant setting in the SUMMIT trial. Investigating 4ICD levels and localization in translational material from such trials may elucidate any mechanistic role for 4ICD in response to neratinib and anti-estrogens.

Other combinations have also shown synergy in preclinical models. The multi-kinase inhibitor regorafenib has displayed synergy with lapatinib in preclinical models of colorectal cancer [137]. In addition, lapatinib enhanced the mammary HER2+ tumor growth-suppression effects of regorafenib and the phosphodiesterase-5 inhibitor sildenafil in vivo [138]. Neratinib has exhibited the same ability to enhance the anti-tumor effects of the regorafenib/sildenafil combination in colon cancer cells in vivo [139].

## 7. Conclusions

Three key properties differentiate the preclinical activities of neratinib and lapatinib: the breadth of targets for neratinib in the HER family of receptors; the greater potency of neratinib versus lapatinib; and the ability of neratinib to irreversibly inhibit EGFR and HER2/4. These properties outline a clear advantage for neratinib in vitro when assessing the cytotoxicity of these TKIs in treatment-naive, treatment-resistant, and *HER*-mutated breast cancer models. The impact of more complete HER-family inhibition extends to the potential impact of neratinib on trastuzumab-mediated ADCC and combination with novel targeted therapies.

The extent to which neratinib’s irreversible, potent, and pan-HER binding in preclinical models influences the clinical profile of this TKI, in particular in relation to that of lapatinib, remains to be determined. To date, the only randomized study comparing neratinib and lapatinib is the phase III NALA trial investigating neratinib plus capecitabine versus lapatinib plus capecitabine in patients with third-line HER2-positive metastatic breast cancer (NCT01808573). Top-line results indicate that treatment with neratinib plus capecitabine resulted in a statistically significant improvement in centrally confirmed progression-free survival and trended positively for overall survival versus lapatinib plus capecitabine [140]. The results of the trial also showed that treatment with neratinib plus capecitabine statistically significantly improved time to intervention for symptomatic brain metastases, a secondary endpoint of the study, versus lapatinib plus capecitabine. Prophylactic treatment for diarrhea has improved the major clinical side effect of neratinib [141], allowing further investigation of neratinib in additional clinical settings for patients with HER2-positive or *HER2-*mutated tumors. Given its unique potent, irreversible, pan-HER kinase inhibitor properties, neratinib has potential to synergize with a variety of molecular targeted therapies, such as trastuzumab, T-DM1, anti-estrogens, Src inhibitors, CDK4/6 inhibitors, HSP90 inhibitors, and HDAC inhibitors, in the treatment-naive or treatment-refractory settings. Indeed, several combination strategies have demonstrated proof of concept in preclinical models and encouraging activity in early clinical trials and further findings from ongoing research with neratinib are awaited with interest.

## Figures and Tables

**Table 1:** Comparison of lapatinib and neratinib.

Characteristic	Lapatinib	Neratinib
Alternative names	GW 282974X; GW 572016; GW-2016; Tykerb^®^ Tyverb^®^ (Novartis International AG, Basel Switzerland)	HKI-272; PB-272; WAY-179272; Nerlynx^®^ (Puma Biotechnology, Inc., Los Angeles, CA, USA)
IUPAC name	N-[3-chloro-4-[(3-fluorophenyl)methoxy]phenyl]-6-[5-[(2-methylsulfonylethylamino)methyl]furan-2-yl]quinazolin-4-amine	(E)-N-[4-[3-chloro-4-(pyridin-2-ylmethoxy)anilino]-3-cyano-7-ethoxyquinolin-6-yl]-4-(dimethylamino)but-2-enamide
ATC classification	WHO: L01XE07 EphMRA: L1H9	WHO: L01XE45 EphMRA: L1H9
Formula	C_29_H_26_ClFN_4_O_4_S	C_30_H_29_ClN_6_O_3_
Molecular weight	Compound: 581.06 g/mol Ditosylate salt: 943.47 g/mol	Compound: 557.05 g/mol Maleate salt: 673.12 g/mol
Receptor binding	Reversible	Irreversible
Route of administration	Oral	Oral
Approved indications	+ Capecitabine in advanced/metastatic HER2+ BC following progression on prior therapy + Letrozole in advanced HER2+ ER+ BC in postmenopausal women	FDA: Extended adjuvant early-stage HER2+ BC following trastuzumab EMA: Extended adjuvant HER2+ ER+ BC following trastuzumab
Mechanism of action	Inhibits EGFR and HER2, preventing autophosphorylation and activation of oncogenic intracellular signaling pathways	Inhibits EGFR, HER2, and HER4, preventing autophosphorylation and activation of oncogenic intracellular signaling pathways
K_d_ IC_50_, nM [7]		
EGFR	2.4	1.1
HER2	7	6
HER4	54	2.4
Elimination half-life	Single dose: 14.2 h [8] Repeated dosing: 24 h	Single dose: 7–17 h [9] Repeated dosing: 14.6 h
Metabolism	Major: CYP3A Minor: CYP2C19; CYP2C8	Major: CYP3A4 Minor: Flavin-containing monooxygenase
ABCB1/P-gp interaction	P-gp inhibitor [10]	P-gp inhibitor [10,11]
Most common AE	Diarrhea [12,13]	Diarrhea [14]

ABCB1, ATP-binding cassette sub-family B member 1; AE, adverse event; ATC, Anatomical Therapeutic Chemical; BC, breast cancer; CYP, cytochrome P450; EGFR, epidermal growth factor receptor; EMA, European Medicines Agency; EphMRA, European Pharmaceutical Market Research Association; ER, estrogen receptor; FDA, Food and Drug Administration; HER, human epidermal growth factor receptor; IC_50_, half maximal inhibitory concentration; K_d_, equilibrium dissociation constant; IUPAC, International Union of Pure and Applied Chemistry; P-gp, P-glycoprotein; WHO, World Health Organization.

**Table 2:** IC_50_ values for lapatinib and neratinib in cell-based assays: therapy-naive and targeted therapy-resistant cell lines.

Cell Line	Breast Cancer Subtype [64,65,66]	Sensitivity to Trastuzumab * [67]	IC_50_ Value ± SD (nM)		Reference
			Lapatinib	Neratinib	
BT474	HER2+/ER+	S	23	3	[57]
			36 ± 15.1	1.9 ± 0.46	[68,69]
			23.9 ± 19.6	0.84 ± 0.11	[53]
			16 ± 11 ^†^	<5	[25]
EFM-192A	HER2+/ER+	S	20.2 ± 0.186	2.38 ± 0.23	[53]
			75 ± 2 ^†^	<5	[25]
UACC-812	HER2+/ER+	S	80.4 ± 32.7	4.03 ± 0.14	[53]
			432 ± 116 ^†^	<5	[25]
MDA-MB-361	HER2+/ER+	S	323.5 ± 50.8	7.36 ± 2.65	[53]
HCC1419	HER2+/ER−	R	63.1 ± 29.7	3.94 ± 0.5	[53]
HCC1569	HER2+/ER−	R	242 ± 8.7	36.24 ± 4.85	[53]
			3550 ± 715 ^†^	<5	[25]
HCC1954	HER2+/ER−	R	582 ± 31	24.73 ± 5.42	[53]
			358 ± 64 ^†^	<5	[25]
JIMT-1	HER2+/ER−	R	1416 ± 371.9	141.5 ± 9.61	[53]
MDA-MB-453	HER2+/ER−	R	260	110	[57]
			6080 ± 825	820 ± 140	[68,69]
			5186.3 ± 1542.8	267.23 ± 22.52	[53]
SKBR3	HER2+/ER−	S	15	3	[57]
			80 ± 17.3	2.26 ± 0.08	[68,69]
			25.9 ± 2.9	1.26 ± 0.24	[53]
			54 ± 8 ^†^	5	[25]
			89	2.1	[70]
SUM-190	HER2+/ER−	D	38 ± 3 ^†^	10 ± 0.0 ^†^	[25]
SUM-225	HER2+/ER−	R	89 ± 52 ^†^	10 ± 0.0 ^†^	[25]
UACC-732	HER2+/ER−	R	3180 ± 939	132.23 ± 19.66	[53]
			2629 ± 480 ^†^	650 ± 370 ^†^	[25]
UACC-893	HER2+/ER−	R	1211 ± 251 ^†^	<5	[25]
MCF7	ER+		2660	350	[57]
T47D	ER+		2870	890	[57]
HCC1937	TNBC		6000	750	[70]
SUM-229PE	TNBC		350	14	[70]
MDA-MB-231	TNBC		9290	1350	[57]
MDA-MB-468	TNBC (EGFR-amplified)		1730	36	[57]
**Resistant cell line models**					
HCC1954-Par	HER2+/ER−	R	273	49	[71]
HCC1954-NR	HER2+/ER−		2700	325	[71]
EFM192A-Par	HER2+/ER+	S	50	6.8	[71]
EFM192A-NR	HER2+/ER+		7970	46.7	[71]
BT474/AZ-P	HER2+/ER+	S	80	0.24	[72]
BT474-LR	HER2+/ER+		3000	8	[72]
BT474/ATCC-P	HER2+/ER+	S	80	0.72	[72]
BT474-LTR	HER2+/ER+		3800	16	[72]

* Sensitivity to trastuzumab based on 2- and 3-dimensional assays as determined by O’Brien et al. [67]. ^†^ Standard error. +, positive; −, negative; D, distinct (resistant in 2-dimensional assay, sensitive in 3-dimensional assay); EGFR, epidermal growth factor receptor; ER, estrogen receptor; HER, human epidermal growth factor receptor; IC_50_, half maximal inhibitory concentration; LR, lapatinib-resistant; LTR, lapatinib- and trastuzumab-resistant; NR, neratinib-resistant; R, resistant; S, sensitive; SD, standard deviation; TNBC, triple-negative breast cancer.

**Table 3:** IC_50_ values for neratinib and lapatinib in cell-based assays: cell lines with *HER2* mutations.

Study and Cell Line	HER2 Mutation	Breast Cancer Subtype [64,65,66]	IC_50_ ± SD (nM)	
			Neratinib	Lapatinib
Bose et al. [75]				
MCF10A	HER2 WT	Non-tumorigenic, epithelial breast	<2	400 ± 60
	G309A		<2	470 ± 50
	V777L		<2	1040 ± 570
	D769H		<2	980 ± 950
	V842I		<2	650 ± 210
	del.755–759		2.1 ± 0.2	660 ± 90
	L755S		15.6 ± 6	>10,000
BT474		HER2+/ER+	<2	32 ± 2
MCF7		ER+	>3000	>10,000
Zuo et al. [76]				
MCF10A	HER2 WT	Non-tumorigenic, epithelial breast	<2	480 ± 50
	K75E		32 ± 8	>10,000
	L768S		<2	1050 ± 480
	V773L		<2	960 ± 380
	R647K		<2	650 ± 370
	I655V		<2	520 ± 420
	K676R		<2	385 ± 270
	Q680R		<2	550 ± 190
BT474	Parental	HER2+/ER+	<2	72 ± 24
	HER2 WT		<2	109 ± 36
	K75E		40 ± 8	1240 ± 460
	L768S		<2	286 ± 110
	V773L		<2	218 ± 85
	R647K		<2	86 ± 40
	I655V		<2	120 ± 52
	K676R		<2	64 ± 36
	Q680R		<2	78 ± 5
MDA-MB-231	HER2 WT	TNBC	410 ± 140	3620 ± 860
	K75E		889 ± 215	>10,000
	L768S		590 ± 165	4980 ± 1110
	V773L		780 ± 184	4980 ± 785
	R647K		472 ± 106	3578 ± 759
	I655V		508 ± 108	3496 ± 808
	K676R		537 ± 124	3730 ± 960
	Q680R		496 ± 98	3778 ± 845
MCF7	Parental	ER+	>3000	>10,000
Cocco et al. [5]				
BT474	L755S	HER2+/ER+	6.7	583.8
SKBR3	L755S	HER2+/ER−	10.2	1424

ER, estrogen receptor; HER, human epidermal growth factor receptor; IC_50_, half maximal inhibitory concentration; SD, standard deviation; TNBC, triple-negative breast cancer; WT, wild type.
